# Variation in abundance of predicted resistance genes in the *Brassica oleracea* pangenome

**DOI:** 10.1111/pbi.13015

**Published:** 2018-05-31

**Authors:** Philipp E. Bayer, Agnieszka A. Golicz, Soodeh Tirnaz, Chon‐Kit Kenneth Chan, David Edwards, Jacqueline Batley

**Affiliations:** ^1^ School of Biological Sciences and Institute of Agriculture The University of Western Australia Crawley WA Australia; ^2^ Plant Molecular Biology and Biotechnology Laboratory Faculty of Veterinary and Agricultural Sciences University of Melbourne Melbourne Vic. Australia; ^3^ Australian Genome Research Facility Melbourne Vic. Australia

**Keywords:** *Brassica oleracea*, resistance genes, RGAs, pangenomics, PAV, Brassicaceae

## Abstract

*Brassica oleracea* is an important agricultural species encompassing many vegetable crops including cabbage, cauliflower, broccoli and kale; however, it can be susceptible to a variety of fungal diseases such as clubroot, blackleg, leaf spot and downy mildew. Resistance to these diseases is meditated by specific disease resistance genes analogs (RGAs) which are differently distributed across *B. oleracea* lines. The sequenced reference cultivar does not contain all *B. oleracea* genes due to gene presence/absence variation between individuals, which makes it necessary to search for RGA candidates in the *B. oleracea* pangenome. Here we present a comparative analysis of RGA candidates in the pangenome of *B. oleracea*. We show that the presence of RGA candidates differs between lines and suggests that in *B. oleracea*, SNPs and presence/absence variation drive RGA diversity using separate mechanisms. We identified 59 RGA candidates linked to *Sclerotinia*, clubroot, and Fusarium wilt resistance QTL, and these findings have implications for crop breeding in *B. oleracea,* which may also be applicable in other crops species.

## Introduction

*Brassica oleracea* is a member of the large and agronomically important Brassicaceae family, which consists of more than 372 genera and 4060 species (The Plant List, 2010). *B. oleracea* species encompass many popular and nutritious vegetable crops including cabbages, cauliflower, broccoli, brussels sprout, kohlrabi and kale. These species are susceptible to a range of diseases including blackleg, clubroot, sclerotinia stem rot, downy mildew and powdery mildew (Channon, 1981; Neik *et al*., 2017; Punithalingham and Holliday, 1972; Voorrips, 1995).

Pathogens use a diverse array of strategies to enter, survive and successfully infect their host. In response to this attack, plants use a two‐branched innate immune system (Jones and Dangl, 2006). Molecules common to many classes of microbes, including non‐pathogens, called pathogen‐ or microbial‐associated molecular patterns (PAMPS or MAMPS) are recognized by the first branch of the immune system, resulting in PAMP‐triggered immunity (PTI), and further colonization can be stopped (Jones and Dangl, 2006). However, pathogens have evolved the capacity to deliver effector molecules or virulence factors to suppress PTI, and such interference can result in effector‐triggered susceptibility (ETS) (Jones and Dangl, 2006; Hammond‐Kosack and Jones, 1996). Specific recognition of effector molecules *in planta* is determined by resistance gene (*R‐*gene) products, and consequently a gene for gene interaction ensues. When both the pathogens’ avirulence (*Avr)* gene and the corresponding plant's *R*‐gene products are present, disease resistance occurs (incompatible interaction). A plant is susceptible if the corresponding *R*‐gene is absent or inactive (compatible interaction; Dangl & Jones, 2001).

There are several classes of *R*‐genes. These classes are defined by their structural motifs (Kruijt *et al*., 2005). The largest class of proteins encoded by *R*‐genes in plant genomes belongs to the nucleotide‐binding site and leucine‐rich repeat (NBS‐LRR) domain‐containing class of proteins (Baumgarten *et al.,* 2003). NBS‐LRR proteins have a variable N‐terminus, which commonly contains a domain with similarity to the *Drosophila* Toll and mammalian interleukin‐1 receptor (TIR) or a coiled coil (CC) sequence. NBS‐LRR domains share a high degree of sequence identity and have a number of conserved motifs, which can be used to identify NBS‐LRR genes (Meyers *et al*., 1999; Neik *et al*., 2017; Wan *et al*., 2012).

*R*‐genes are grouped into resistance gene analogs (RGAs) with pattern‐recognition receptors (PRRs; Sekhwal *et al*., 2015). PRRs are classified into two groups: surface‐localized receptor‐like protein kinases (RLKs; Walker, 1994) and membrane associated receptor‐like proteins (RLPs). RLKs and RLPs are a large group of proteins that are necessary for regular plant development (Morris and Walker, 2003) but are also necessary in plant disease resistance (Kruijt *et al*., 2005). RLKs carry a cytoplasmic‐kinase domain while RLPs carry a short cytoplasmic tail. In tomato, it has been shown that RLKs and RLPs interact: RLKs act as receptors which mediate downstream signalling by way of binding RLPs (Liebrand *et al*., 2013).

The increasing availability of plant genomes allows for the *in silico* analysis of gene families, such as NBS‐LRRs and PRRs. Genome‐wide analysis of NBS‐LRRs has been performed in numerous plant species (Ameline‐Torregrosa *et al*., 2008; Gu *et al*., 2015; Jupe *et al*., 2012; Kang *et al*., 2012; Lozano *et al*., 2012, 2015; Meyers *et al*., 2003; Seo *et al*., 2016; Singh *et al*., 2015; Wei *et al*., 2013; Zheng *et al*., 2016; Zhou *et al*., 2004). Recently, there has been analysis of NBS‐LRRs in several *Brassica* species, including *B. rapa*,* B. napus* and *B. oleracea* (Alamery *et al*., 2018; Chalhoub *et al*., 2014; Golicz *et al*., 2016; Lv *et al*., 2015; Mun *et al*., 2009; Sarris *et al*., 2016; Shao *et al*., 2016; Wu *et al*., 2014; Yu *et al*., 2014; Zhang *et al*., 2016a,b). RLKs and RLPs have been comprehensively mined in wild strawberry (Li *et al*., 2017) and characterized in tomato (Kawchuk *et al*., 2001), *Arabidopsis thaliana* (Wang *et al*., 2008), rice (Fritz‐Laylin *et al*., 2005) and poplar (Petre *et al*., 2014). This analysis has demonstrated that different genomes contain different RGAs and that there is a variation in RGA content between different lines.

It has been shown in several plant species that groups of *R*‐genes and PRRs cluster closely together within the genome. For example in *B.  napus* several *Leptosphaeria maculans*‐specific *R*‐genes cluster closely together (Delourme *et al*., 2004), as do clubroot resistance genes in *B.  rapa* (Kato *et al*., 2013). This close linkage is important in generating novel resistance by recombination (Hulbert *et al*., 2001), where intragenic crossover events lead to new motif combinations.

A second mechanism that introduces diversity is transposable element (TE)‐mediated rearrangement, diversification and duplication. A TE may also change gene expression by inserting itself into genes or by recruiting repressive methylation and therefore changing expression in nearby genes (Masson *et al*., 1987; McClintock, 1956), and *R*‐gene clusters have been shown to be associated with TEs in rice (Song *et al*., 1997) and barley (Wei *et al*., 2002).

Analysis of the *B. oleracea* pangenome has identified abundant structural variation [presence absence variants (PAV) and copy number variants (CNV)]. Golicz *et al*. (2016) showed that 18.7% of the 61 380 *B. oleracea* genes in the pangenome were not present in all lines. In addition, many of these dispensable genes are related to agronomic and other important traits, including disease resistance, suggesting that PAVs may be important for the breeding of improved *Brassica* crops. The gene‐for‐gene interaction in disease resistance drives a molecular arms race between pathogen and host, and natural selection drives pathogens to either dispense of or diversify its array of effectors, while host plants are required to combat this through *R*‐gene duplication and diversification (Jones and Dangl, 2006; Dangl and Jones, 2001). In order to better understand this process, we have identified candidate RGAs in the pangenome of *B. oleracea*. We investigate which are core or dispensable and determine whether those in clusters are more likely to be lost or conserved. We then link the PAV status of RGAs with the presence or absence of TEs showing that TEs are strongly associated with variability in RGAs.

## Results and discussion

### Genome‐wide distribution of RGA candidates

**Figure 1:**
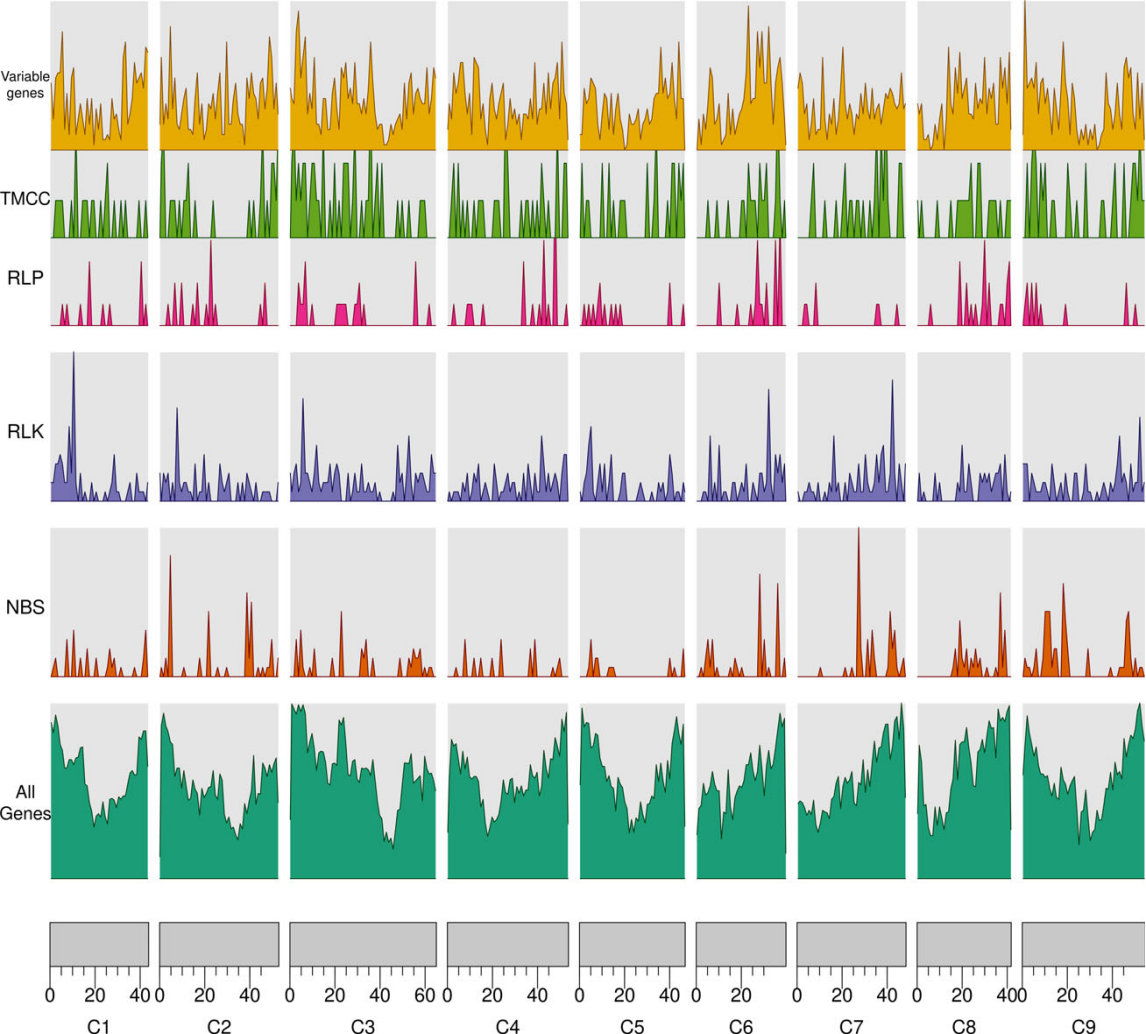
Density of genes compared with the density of NBS, RLK, RLP as well as variable genes.

A total of 1989 RGA candidates were identified in the *B. oleracea* pangenome (Figure 1, Table S1). The largest class of resistance gene candidates was RLKs (901), followed by NBS‐LRR genes (556; Table 1). TX (TIR domain with unknown domain) and TNLs (TIR domain, NB‐ARC domain and Leucine‐rich‐repeat domain) were the largest subclass within the NBS‐LRR genes (129 and 123). The RGA candidate density per pseudomolecule was roughly similar to all pseudomolecules (average: 3.7 RGA candidates per Mbp ranging from 2.8 on pseudomolecule C5 to 4.3 on C9), though the additional contigs which are not contained within the reference assembly harboured more NBS‐LRR and RLK than RLP candidate genes (121, 79, and 54 respectively).

**Table 1:** Count of resistance gene classes in the *Brassica oleracea* pangenome

Class	C1	C2	C3	C4	C5	C6	C7	C8	C9	Unplaced scaffolds	Total in reference genome	Additional contigs in pangenome	Total
CN	2	2	0	1	1	2	3	3	0	2	16	11	27
CNL	5	3	6	6	3	5	4	6	5	4	47	9	56
NBS	2	5	4	2	2	5	3	4	5	8	40	25	65
NL	7	7	8	2	3	5	7	10	24	4	77	42	119
TN	1	5	2	2	1	12	2	3	4	1	33	4	37
TNL	8	25	18	6	4	7	15	9	24	5	121	8	129
TX	9	8	13	6	3	7	15	5	21	18	105	18	123
Total NBS‐LRR	34	55	51	25	17	43	49	40	83	42	439	117	556
RLK LRR	32	31	65	27	32	32	41	17	32	12	321	13	334
RLK Other	51	44	74	65	38	43	57	42	68	19	501	66	567
Total RLK	83	75	139	92	70	75	98	59	100	31	822	79	901
RLP LRR	12	16	22	23	12	22	7	20	12	10	156	54	210
RLP Other	0	1	0	0	1	0	0	1	0	0	3	0	3
Total RLP	12	17	22	23	13	22	7	21	12	10	159	54	213
Other	1	2	1	1	1	2	4	2	0	0	14	4	18
Total	152	179	268	177	129	165	189	146	235	89	1729	260	1989
Total per Mbp	3.47	3.38	4.12	3.29	2.75	4.14	3.91	3.50	4.30	2.13	3.87	2.64	3.65

NBS‐LRR genes have been previously mined in *B. oleracea* where 239 NBS‐LRR were identified (Yu *et al*., 2014), around half the number identified in this study. We used a newer annotation and assembly as it has been shown that older *B. oleracea* annotations contain incomplete or misannotated *R*‐genes (Lv *et al*., 2014). Such analyses of *R*‐gene identification can be influenced by the quality of the genome assembly. Errors in the genome assembly or gene prediction can lead to wrongly predicted numbers of *R‐*genes. Other methods exist to gain insight into the number of *R*‐genes which do not rely on reference genomes, such as ReNSeq for NBS‐LRR identification (Jupe *et al*., 2013). We also used an improved RGA candidate prediction pipeline which compared RGA candidates with a larger set of known *R*‐genes, while the previous study discarded *R*‐gene candidates that did not align with a *B. oleracea* specific NBS profile.

We found that the largest class of RGA candidates was RLKs, which is consistent with observations in other plants such as wild strawberry and cotton (Chen *et al*., 2015; Li *et al*., 2017). The larger number of RLK than RLP and NBS‐LRR genes could be due to a greater diversity of roles of these genes. In the Brassicaceae, RLK genes have been implicated in a variety of regular developmental mechanisms such as self‐incompatibility (Takayama and Isogai, 2003) so they are not necessarily involved in resistance. There is no such functional diversity in other RGA classes such as NBS‐LRR genes (McHale *et al*., 2006).

We found that resistance genes were unevenly distributed along the pseudomolecules (Table 1) which fits prior observations including in *B. oleracea* (Golicz *et al*., 2016), *B. napus* (Chalhoub *et al*., 2014), and other plant species such as rice (Rice Chromosomes 11 and 12 Sequencing Consortia, 2005). This uneven distribution is likely due to recent tandem gene amplifications and segmental duplications (Rice Chromosomes 11 and 12 Sequencing Consortia, 2005).

We compared the counts of RGA candidates within the different lines based on presence/absence results. The wild type relative *B. macrocarpa* contains the highest number of RGA candidates (1495), 45 more than the average total of the domesticated *B. oleracea* lines and 93 more than the reference cultivar TO1000 (Table 2). Six RGA candidates appear only in TO1000 (two TX, two TN and two NL), no other RGA candidates appear in only one line. TO1000 carried the lowest number of RGA candidates in all categories except TN, TNL and TX. Of the RGA candidate genes, 1231 were present in all lines (core) and 167 (12%) were variable (lost in at least one line). RLKs and CNLs showed the lowest percentage of variable genes (4.5% and 8.5% respectively), while NL and NBS showed the highest number of variance (31.2% and 32.4%). The high percentage of variable genes that are incomplete (NL, CN, TN) could mean that these are pseudogenes that the genome can afford to lose without consequences in the form of lost resistance.

**Table 2:** Count of RGA candidates per line and RGA class

Name	Line	CN	CNL	NBS	NL	RLK	RLP	TN	TNL	TX	Total
Early Big	Broccoli	19	48	46	90	834	169	31	108	105	1450
AC498 (Gower DH line)	Brussels Sprouts	17	53	43	86	850	181	30	115	96	1471
Badger Inbred 16	Cabbage1	15	52	40	85	844	173	30	117	97	1453
HRIGRU009617 DH3	Cabbage2	20	50	39	90	846	174	29	113	100	1461
BOL909	Cauliflower1	19	48	37	88	841	171	30	117	97	1448
CA25 (Nedcha DH line)	Cauliflower2	18	52	41	84	847	172	30	110	93	1447
ARS_18 (Arsis DH)	Kale	18	53	38	86	846	178	29	115	99	1462
HRIGRU011183 DH1	Kohlrabi	17	51	39	87	842	174	28	113	99	1450
*B. macrocarpa*	*B. macrocarpa*	21	52	41	97	862	186	30	110	96	1495
TO1000 DH3	TO1000	14	47	34	79	821	157	32	119	99	1402

Interestingly, there were more NBS‐LRR than RLK and RLP genes in the additional pangenome non‐reference contigs, indicating that NBS‐LRR genes show a greater variability than RLK and RLP genes. Based on PAV data, these RGA candidates are differently distributed between the nine individuals on which the pangenome was based. As expected, the wild relative *B. macrocarpa* carried the greatest number of RGA candidates and these may have been lost in domesticated lines during domestication. *B. macrocarpa* shows partial leaf and moderate stem resistance to *Sclerotinia sclerotiorum* infection (Taylor *et al*., 2018) to which all *Brassica* plants are susceptible, and this may be linked with the additional RGA candidates. The kale‐like reference cultivar TO1000 showed the lowest number of RGA candidates indicating that in searching for RGAs it is best to not focus on the reference genome alone.

### Physical cluster distribution

We allocated RGA candidates to physical RGA‐gene‐rich clusters where *R*‐genes are within 10 genes of each other, and found 744 out of 1729 (43%) of RGA candidates reside within physical clusters. The percentage of physically clustered genes varied between RGA classes. For NBS‐LRR genes, 310 out of 453 (68%) genes were located in physical clusters, while 339 out of 822 (41%) RLK genes and 53 out of 159 (33%) RLP were located in physical clusters. In most plants it has been shown that RGA candidates locate within RGA‐gene‐rich clusters. In *Arabidopsis*, 113 out of 159 (71%) NBS‐LRR genes are located within RGA‐gene‐rich clusters (Guo *et al*., 2011), and in rice, 76% of NBS‐LRR genes are located within RGA‐gene‐rich clusters (Zhou *et al*., 2004). More recently, three *Yr* genes conferring resistance to wheat yellow rust where found in wheat, all of which are located within the same cluster (Marchal *et al*., 2018).

The RGA candidate density was compared with available PAV data. There were more variable NBS‐LRR genes within physical clusters than outside of clusters (87, 18) and more core NBS‐LRR genes within physical clusters than outside of clusters (219, 111). A chi‐square test was used to test for independence of these two classes and they were not equally distributed (χ^2^ = 9.6, *P* < 0.05). This indicates that the PAV status of NBS‐LRR genes and the physical cluster status is strongly linked, suggesting that the presence of a gene within a cluster may protect it from being lost. NBS‐LRR genes are often located in physical clusters containing many perfect copies (Michelmore and Meyers, 1998). However, membership in such a cluster does not always protect from loss, as deletions within *R*‐gene clusters have been described in the melon genome, where a 146 kb deletion was affecting a 23 *R*‐gene cluster with two more structural variants affecting *R*‐genes (Sanseverino *et al*., 2015).

### Sequence‐based clustering of RGA candidates

The RGA candidates were clustered based on the sequence identity to find the number of allelic variants describing the true extent of RGA diversity in the 10 lines. At a minimum identity of 70%, we found 280 clusters with 716 members in total, ranging from 2 to 7 members. Of the clustered RGA candidate classes, 188 were RLKs, 133 were NBS‐LRR, 26 were RLPs and 972 RGA candidates remained as unclustered singletons. Most of the largest clusters contained only RLKs as almost perfect copies from different locations in the genome (cluster 0: 7 RLKs, cluster 1: 6 RLKs, cluster 2: 2 NBS, 2 TNLs, 1 NL, 1 TX, cluster 3: 1 NBS, 1 TNL, 1 CNL, 2 OTHER, 1 TX, cluster 4: 6 RLKs; Figure S1).

Most genes within clusters were present in all lines (563 core, 153 variable). The 10 largest clusters containing only RLKs consisted of mostly core genes (median 100% core, average: 96% core). The clusters were classified based on whether they contained only RLK, only RLP, only NBS‐LRR genes, or a mixture of these three. All the clusters contained on average and median 79% core genes, independent of type.

The average sequence identity of all clusters was 80.4% indicating relatively high divergence between *R*‐gene paralogs. In *Arabidopsis*, there are very few *R*‐gene paralogs which show high sequence identity, which we also observe, with the majority of *R*‐genes showing no perfect identity (Bergelson *et al*., 2001). This is likely due to the positive selection acting on these *R*‐genes.

### RGA candidates and transposable elements

**Figure 2:**
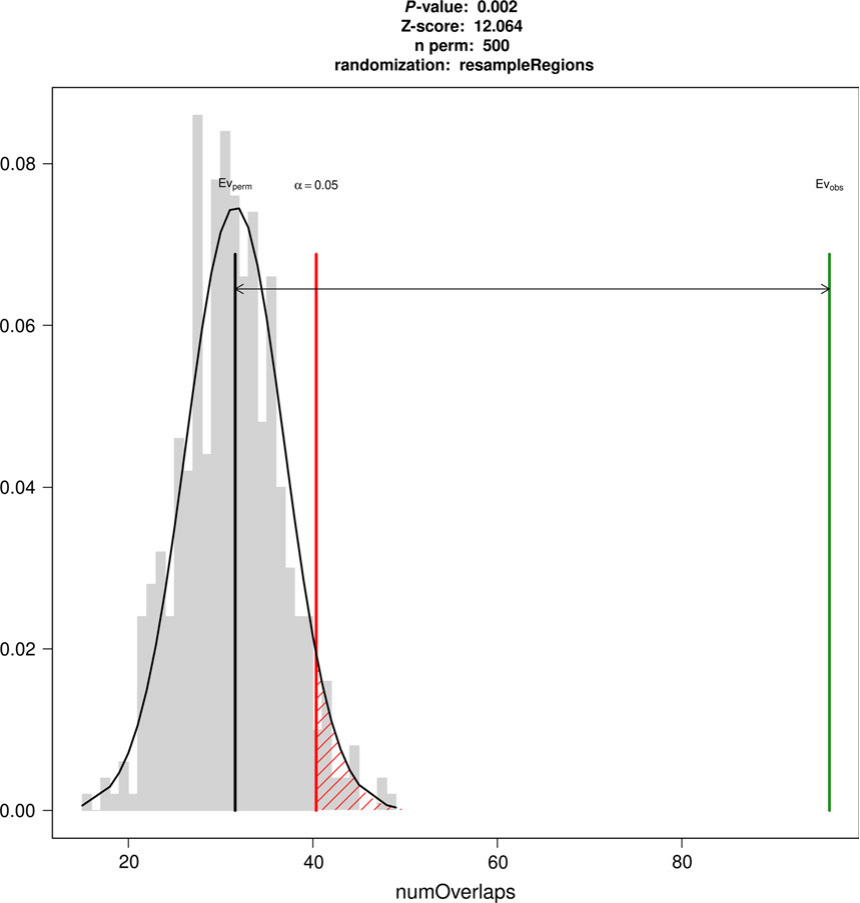
Expected (Ev_perm_) and observed (Ev_obs_) overlaps between NBS genes and PAV genes showing that the number of overlaps is higher than expected.

We tested the hypothesis whether resistance genes and variable genes overlap more often than expected when compared with all other genes. To this end, we tested for physical overlap and physical distance‐based association between resistance gene classes and variable genes. NBS‐LRR genes showed the greatest variability of all classes of RGAs based on the strongest, statistically significant overlap association with a *Z*‐score of 11 (*P* < 0.005; Figure 2, Table S2). RLK genes, on the other hand, showed significantly less variability than expected (*P* < 0.05, Table S2, Figure S2).

We searched for RGA candidates which are lost or retained in pairs, as a proxy for selection pressure. *R*‐gene pairs that are lost together but are in close proximity may have been lost due to chance, while *R*‐gene pairs that are distant from each other but have been lost together may hint at a selection pressure impacting gene conservation, as has been observed with homeologous gene pairs where copies subfunctionalize (Adams *et al*., 2003).

Of the 168 variable RGA candidates, 59 pairs [118 (70%) RGA candidates] were present or absent in the same individuals. Of these, 35 RGA candidates were located on the same pseudomolecule, and a further 11 pairs were located within 10 Kbp of each other. These 11 pairs may have been lost due to chance alone. Another 24 pairs were located on different pseudomolecules. These pairs were mostly evenly distributed along the pseudomolecules with a maximum of three pairs between RGA candidates on C8 and C9. In *B. oleracea,* specific *R*‐genes were previously associated with hybrid lethality (HL; Xiao *et al*., 2017), where two interacting loci cause hybrid failure, eventually leading to speciation (Orr, 1996). While none of the RGA candidates lost in tandem were linked to HL before in *B. oleracea* (Xiao *et al*., 2017), it may explain some of the genes lost in tandem here.

We investigated whether the RGA candidate and PAV association is dependent on the position in the genome, or whether the association is class‐dependent alone. The association between NBS‐LRR candidate genes and PAV, and the association between TEs and PAV genes are highly context and position dependent since the association measured by the *Z*‐score falls after shifting positions for more than 3 Kbp (Figure S3).

We searched for elements, such as TEs and simple repeats, in surrounding regions and checked for the distance association between PAVs and TEs. In our data, there was no association between PAVs and simple repeats; however, the average distance between TEs and PAVgenes is lower than expected (*P* < 0.05, Figures S4 and S5). This association also holds up for the mean distance between TE and NBS‐LRR genes, but not for RLK genes, which fits with the previous observation that NBS‐LRR genes are the most variable class of all the RGAs. TE activity has previously been linked with *R*‐genes (Hulbert *et al*., 2001). In rice, it has been shown that an inactive rice blast resistance gene has undergone refunctionalization due to the recruitment of a retrotransposon (Hayashi and Yoshida, 2009). In *A. thaliana,* a complex regulatory system involving TEs has been described, where a transposon insertion in an intron of *RPP7* (resistance to downy mildew) controlled by *EDM2* results in two different *RPP7* isoforms which are both important in the plant's pathogen resistance (Tsuchiya and Eulgem, 2013). Therefore, TEs seem to have an important role in generating novel disease resistance in plants. TEs also play a role in presence/absence variation (PAV). During TE transposition in maize, unrelated genes located nearby can be captured and relocated (Lai *et al*., 2005) causing PAV.

### SNP analysis

To examine the association between SNPs and RGAs, we compared the 4 815 081 SNPs called by (Golicz *et al*., 2016) with our RGA candidates. Predicted functional changes were analysed within the RGA candidates. Of the SNPs positioned within RGA candidate coding sequences, 434 were predicted to be high impact, 17 109 moderate impact and 16 515 low impact. Of the high impact SNPs, 267 introduced premature stop codons, 33 led to the loss of stop codons, 23 lead to lost start codons, and finally 53 splice acceptor variants and 58 splice donor variants were introduced. There were more high impact SNPs in RGA candidate genes than in non‐RGA candidate genes, with 434 high impact SNPs for 1729 RGA candidate genes (ratio: 0.26), and only 9860 high impact SNPs for 59 651 non‐RGA genes (ratio: 0.16). In *Arabidopsis*,* R*‐genes are known to amass unusually large numbers of non‐synonymous SNPs, producing new allelic variants of *R*‐genes (Bakker *et al*., 2006) which corresponds with our observations.

**Figure 3:**
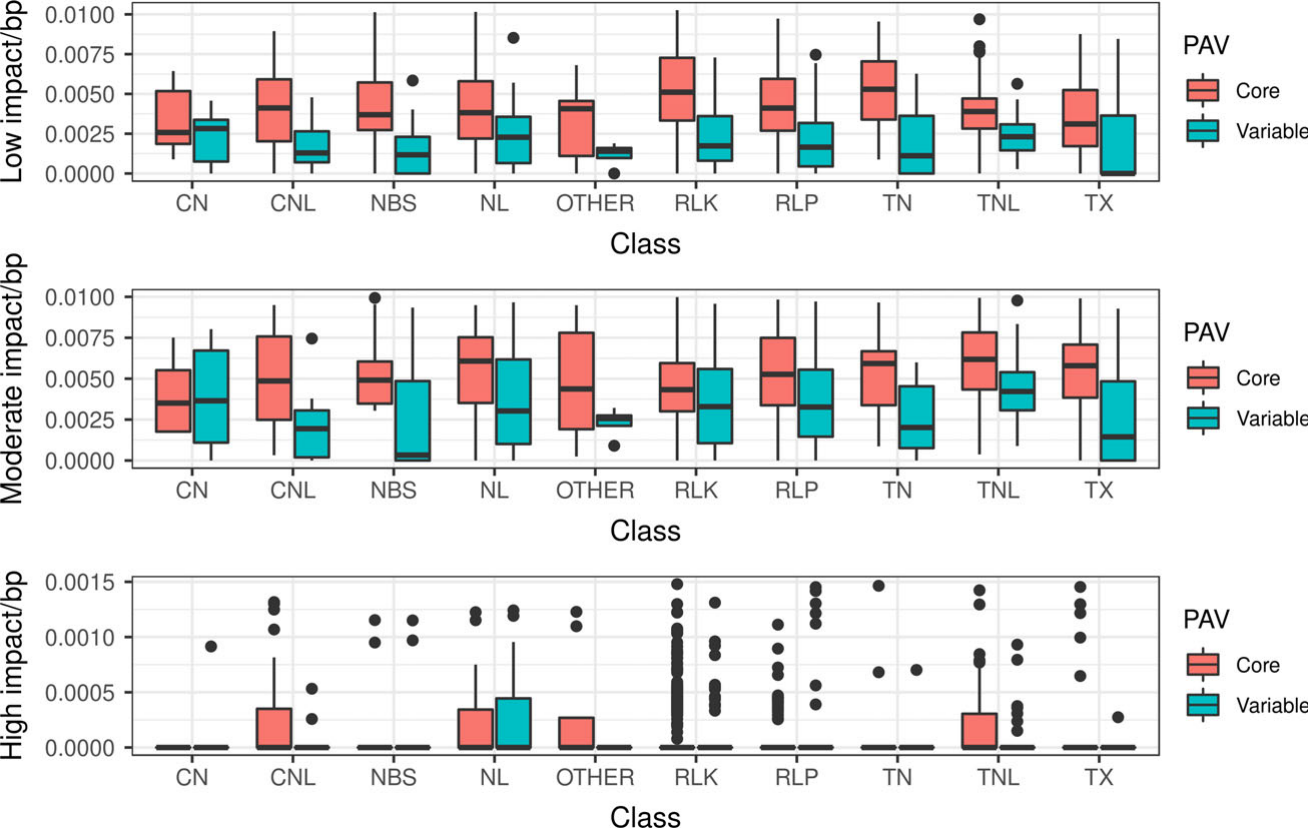
High impact, moderate impact and low impact SNPs per base pair compared with RGA class and presence/absence status.

Resistance genes analogs located on the extra pangenome contigs contained fewer SNPs than RGAs located on pseudomolecules. Out of 1729 RGAs on pseudomolecules, 23 contained no SNPs (1%), while out of 261 RGAs on the extra pangenome contigs, 97 contained no SNPs (37%). RLK genes in particular showed more low impact variants than the other RGA classes (Figure 3), with similar patterns in upstream and downstream variants (Figure S6).

We expect to see a lower SNP density in the non‐reference contigs since these contigs exist in only a few individuals. On average, core genes had more low and moderate impact SNPs than variable genes (average: 12 in core, four in variable genes). Core genes and variable genes had almost identical numbers of high impact SNPs (average 0.25 in both cases). This comparison is confounded by the lower SNP density in variable genes (35 187 SNPs in core, 3665 in variable genes). In *Arabidopsis* populations, the nucleotide diversity in *R*‐genes showing presence/absence variation is much lower than in core *R*‐genes (Shen *et al*., 2006), indicating two different mechanisms of selection. This may be the case in *B. oleracea* as well.

### Linking SNPs and PAV

The interplay between variation generated by SNPs and variation generated by PAVs was assessed. In 303 RGA candidates, of which 132 were NBS‐LRRs, 131 were RLKs, and 40 RLPs, likely loss of function causing SNPs were identified. Interestingly, even though there were more RLKs found in the pangenome, the percentage of NBS‐LRR genes with loss of function SNPs was higher than in RLKs, again indicating that NBS‐LRRs mutate faster as observed with higher PAV in NBS‐LRRs (Figure S6). This discrepancy could also be due to different roles in RLKs past disease resistance.

Of the RGA candidates carrying at least one loss of function SNP, 66 genes were variable and 237 were present in all lines, which fits with the previous observation that variation generated by SNPs is mostly distinct from variation generated by PAV.

Resistance genes‐analog class and PAV was linked significantly with the number of moderate and low impact SNPs (Chi‐squared test, *P* < 0.05). High impact SNPs were only statistically significantly linked with RGA class and not with PAV status. This indicates that PAV and SNP based variability act separately from each other in *B. oleracea* RGA candidates, as has been observed in *Arabidopsis* (Tan *et al*., 2012). There seem to be two different paths to increase RGA gene diversity, which seem to work mostly exclusive from each other—either a gene collects a loss of function mutation, or it is lost entirely. Why these two mechanisms do not significantly overlap remains to be determined.

### Linking known QTL and *R*‐genes

The RGA candidate positions were compared with known quantitative trait loci (QTL) for *Sclerotinia,* clubroot and Fusarium wilt resistance to assess possible biological functions. Pseudomolecule positions were predicted for 32 out of 49 QTL markers, leading to 12 out of 18 reported QTL with positions in the v2.1 assembly. These QTL covered between 0.2 and 34.6 Mbp (average: 9.8 Mbp) on five pseudomolecules with a total of 10 863 genes, out of which 297 genes (2.7%) were RGA candidates (Table 3). Ignoring the four QTL larger than 10 Mbp resulted in 2528 genes underlying the QTL, out of which 59 (2.3%) were RGA candidates.

**Table 3:** Reported QTL for Sclerotinia and black rot resistance, their position in the *Brassica oleracea* v2.1 assembly and RGA candidates contained therein

QTL name	Resistance	Publication	Pseudomolecule	Start (Mbp)	End (Mbp)	Length (Mbp)	RGA candidate classes	Core genes percentage
BRQTL‐C1_2 (BoRSdcaps1‐13 ‐ BoEdcaps4)	Black rot	Lee *et al*. (2015)	C1	18.2	37.1	18.9	CN 2, CNL 6, NL 6, TNL 2, TX 4, RLK 30, RLP 4	54 core (100% core)
BRQTL‐C1_2 (BoRSdcaps1‐14 ‐ BoEdcaps4)	Black rot	Lee *et al*. (2015)	C1	19.6	37.1	17.5	CN 1, CNL 3, NL 3, TNL 1, TX 2, RLK 15, RLP 2	27 core (100% core)
BRQTL‐C1_2 (BoESSR089 ‐ BoEdcaps4)	Black rot	Lee *et al*. (2015)	C1	32.1	37.1	5	RLK 3	3 core (100% core)
QTL‐1 (BoCL3135s ‐ BoCL5545s)	Black rot	Kifuji *et al*. (2013)	C2	1.9	6.3	4.4	CNL 1, NL 1, TNL 13, RLK 9, RLP 2	7 variable, 19 core (73% core)
qLR10‐3 (SWUC177 ‐ BoGMS1032)	*Sclerotinia sclerotiorum*	Mei *et al*. (2013)	C7	12.1	46.7	34.6	CN 3, CNL 4, NBS 3, NL 7, OTHER 4, TN 2, TNL 13, TX 14, RLK 85, RLP 3	16 variable, 122 core (88.5% core)
qLR10‐6 (Ol10D08 ‐ SWUC731)	*Sclerotinia sclerotiorum*	Mei *et al*. (2013)	C9	3.8	4.2	0.5	RLK 1, RLP 2	2 variable, 1 core (33% core)
qLR09‐6 (SWUC731 ‐ SWUC700)	*Sclerotinia sclerotiorum*	Mei *et al*. (2013)	C9	3.8	4.4	0.7	RLK 3, RLP 2	2 variable, 3 core (60% core)
qSR10‐4 (SWUC711 ‐ SWUC700)	*Sclerotinia sclerotiorum*	Mei *et al*. (2013)	C9	4.2	4.4	0.2	RLK 2	2 core (100% core)
qLR09‐5 (SWUC658 ‐ SWUC635)	*Sclerotinia sclerotiorum*	Mei *et al*. (2013)	C9	6.3	7.3	1	CNL 1, NL 1, RLP 1	3 core (100% core)
qLR10‐5 (SWUC679 ‐ SWUC635)	*Sclerotinia sclerotiorum*	Mei *et al*. (2013)	C9	6.8	7.3	0.5	N/A	N/A
CRQTL‐GN_1 (comp7993 ‐ BoRSdcaps2‐10)	Clubroot	Lee *et al*. (2016)	C2	13.5	43.5	30	CN 2, NBS 3, NL 2, OTHER 1, TN 5, TNL 10, TX 7, RLK 37, RLP 9	7 variable, 69 core (91% core)
CRQTL‐GN_2 (BoRSdcaps3‐2 ‐ BoRSdcaps3‐4)	Clubroot	Lee *et al*. (2016)	C3	0.8	5.5	4.7	TN 1, TNL 6, TX 2, RLK 10, RLP 3	7 variable, 15 core (68% core)

**Figure 4:**
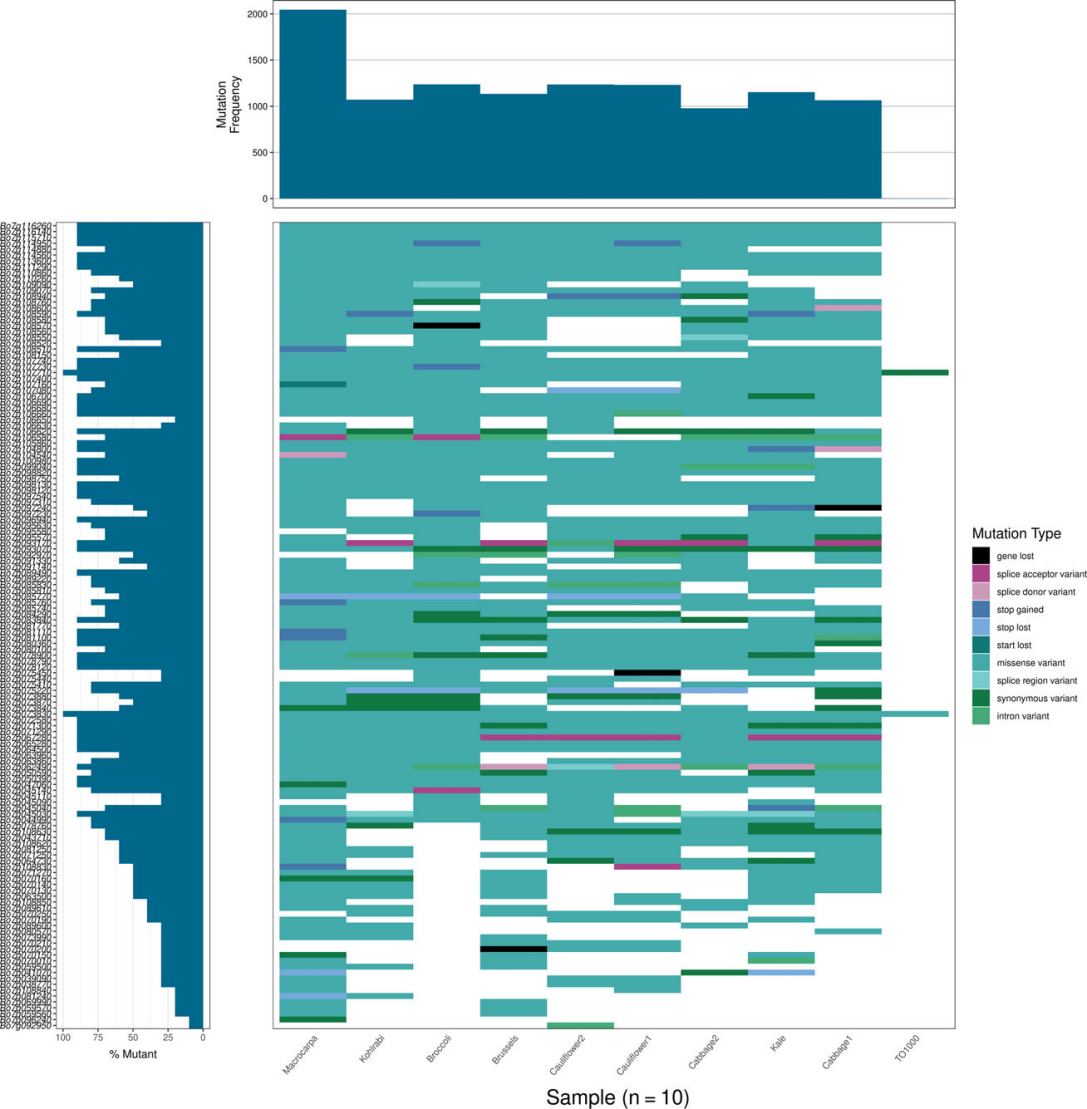
Waterfall plot of the *Sclerotinia* resistance‐linked QTL qLR10‐3 (SWUC177 ‐ BoGMS1032). Gene order is determined by position in the reference assembly.

The majority of the 59 RGA candidates in smaller QTL linked with *Sclerotinia,* black rot, and clubroot are present in all lines (43 core, 16 variable) indicating that these resistances show stable inheritance, which make them valuable targets for plant breeders. The RGA classes showed different levels of variability—27 of the 28 RLKs were core with only one RLK being variable, while 10 TNLs were variable with nine TNLs being present in all lines, indicating that RLKs are more stable than TNLs. A waterfall plot of the *Sclerotinia* resistance‐linked QTL qLR10‐3 (SWUC177 ‐ BoGMS1032) was produced to show the mutational load of RGA candidates located within the QTL candidate region in all 10 individuals (Figure 4). As expected, *B. macrocarpa* showed the highest mutational load. Two genes (Bo7g107710 and Bo7g073830) showed mis‐sense variants in almost all individuals. Very few low impact variants such as synonymous variants were observed in this QTL with the majority of variants being mis‐sense. Variants resulting in lost stop codons were always shared in at least two individuals indicating that this mutation is not random. The large number of mis‐sense variants indicates that this QTL region is under positive selection pressure.

We have shown that there is a large amount of SNP and PAV variability within this QTL region, with some genes being completely lost in a few individuals. Pinpointing the actual cause of resistance will require looking at all aspects of variability in this region in several diverse individuals.

### Linking known *R*‐genes

Lv *et al*. (2014) reported the TNL Fusarium wilt resistance gene *FOC1* to be Bol037156 on pseudomolecule C7 (38.8 Mbp) in *B. oleracea* in the first *B. oleracea* annotation (Liu *et al*., 2014). The short LRR‐domain carrying Bol037156 was re‐annotated into a longer version named ‘re‐Bol037156’ carrying TIR‐NBS‐LRR domains, which segregated as expected in a resistant and susceptible population. We used blastp to search for Bol037156 in the current v2.1 *B. oleracea* assembly and found the best hit in Bo7g104800 on pseudomolecule C7 (score: 325, e‐value 1e^−102^). In the new v2.1 results, this gene is a much longer TNL (2646 amino acids compared with the original 203 amino acids) indicating that in the Liu *et al*. (2014) assembly this gene annotated incorrectly. This fits with the longer, but possibly still too short 1.3 kaa reassembly of re‐ Bol037156 presented in (Lv *et al*., 2014) and indicates that the gene was assembled correctly in v2.1.

Of the 17 *FOC1* linked markers, 13 map to C7 from 40.0 to 42.9 Mbp, which supports the notion of Bo7g104800 being *FOC1*, since Bo7g104800 is located at 40.3 Mbp on C7. Bo7g104800 is present in all 10 lines, however SNPs introduced premature stop codons in ARS_18 (Kale) and Badger Inbred 16 (Cabbage1) which suggests loss of function and subsequent Fusarium wilt susceptibility.

The 34.6 Mbp QTL qLR10‐3 for *Sclerotinia* resistance also contains Bo7g104800. In the light of no published literature linking *Sclerotinia* resistance and Fusarium wilt resistance it is more likely that this overlap is accidental due to the large QTL region.

## Conclusions

Here we have described different modes of selection in RGA candidates in *B. oleracea*. We showed different selection pressures acting on SNPs and PAV for different RGA classes in *B. oleracea*, especially for RLKs and TNs. We have also observed that genes within physical clusters are more likely to be variable, and that similar copies of the same RGAs are retained with a rate of around 80%. We show that there are two mechanisms operating separately from each other in *B. oleracea* that generate diversity in RGAs—one via SNPs and one via PAVs. We have identified 37 RGA candidates within QTL regions associated with *Sclerotinia* and black rot resistance, and these candidates may inform future breeding efforts in *B. oleracea*. We have identified RGA candidates in the pangenome which are not present in the single reference assembly, showing that a pangenome is required to describe the full extent of genes present in the species, as well as necessary for candidate gene identification for breeding of improved cultivars.

## Experimental procedures

The RGAugury pipeline (version 2017‐10‐21; Li *et al*., 2016) was used to predict NBS, RLK, and RLP candidate genes in the *B. oleracea* TO1000 v2.1 annotation downloaded from Ensembl Genomes Release 37 (Kersey *et al*., 2017; Parkin *et al*., 2014) as well as in the *B. oleracea* pangenome downloaded from (http://brassicagenome.net) (Golicz *et al*., 2016). PAV, TE and SNP data for the *B. oleracea* genome and pangenome extra contigs were downloaded from http://brassicagenome.net.

NBS‐LRR were classified based on presence or absence of specific domains: Proteins carrying only an NB‐ARC domain were classified as NBS, proteins carrying TIR, NB‐ARC, and Leucine‐Rich‐Repeat domains were classified as TNLs, or TN if the Leucine‐Rich‐Repeat domain was missing. Proteins carrying Coils, NB‐ARC, and Leucine‐Rich‐Repeat domains were classified as CNLs, or CN if the Leucine‐Rich‐Repeat domain was missing, or NL if the Coils domain was missing. Proteins carrying a TIR domain with additionally unknown domains were classified as TX, while proteins carrying TIR and Coils but not NB‐ARC domains were classified as OTHER.

Resistance genes‐analog candidates were clustered into sequence‐based clusters using CD‐HIT v4.6.8‐2017‐1208 (minimum identity *c*: 0.7; Li and Godzik, 2006). Multiple sequence alignments of each cluster were drawn using MUSCLE v3.8.1551 (Edgar, 2004) and BOXSHADE v3.21 (https://embnet.vital-it.ch/software/BOX_form.html).

The R‐package regioneR v1.8 (Gel *et al*., 2016; R Core Team, 2016) was used to test resistance genes and genes exhibiting PAV and transposable elements for association using 500 permutations. For PAV association, the evaluation function numOverlaps was used to check whether the number of gene overlaps is higher than expected. For TE association, the evaluation function meanDistance was used since we do not expect TEs to overlap with RGA candidates due to repeats having been masked during the annotation process. The R‐package karyotypeR v1.2.2 (Gel and Serra, 2017) was used to plot gene densities.

Resistance genes‐analog‐gene‐rich‐physical clusters were mined from the genome by comparing all resistance gene candidates located on pseudomolecules. Resistance gene candidates were merged into RGA‐gene‐rich clusters if there was at least one other resistance gene within 10 upstream or 10 downstream genes using a Python 3 script (makeRGeneClusterAnalysis.py). Physical clusters and presence/absence status was compared using Pearson's Chi‐squared test with Yates’ continuity correction as implemented in R v3.4.2 (R Core Team, 2016).

The SNPs called by (Golicz *et al*., 2016; available at http://brassicagenome.net/databases.php) were compared with the RGAs using SnpEff v4.3T (Cingolani *et al*., 2012). Since there were more core than variable genes and since core genes were longer than variable genes (Golicz *et al*., 2016) the counts of low, moderate, and high impact SNPs were normalised by dividing by the total length of all exons per gene in order to account for very long and very short genes. Two‐way ANOVA as implemented in R v3.4.2 (R Core Team, 2016) was used to check whether the variation in low, moderate, high, and upstream and downstream variants could be explained by the presence/absence status or by the RGA class.

Known *Sclerotinia,* clubroot and black rot resistance‐linked QTLs were collected from (Kifuji *et al*., 2013; Lee *et al*., 2015, 2016; Mei *et al*., 2013) and 49 marker sequences were collected from (Iniguez‐Luy *et al*., 2009; Izzah *et al*., 2014; Lee *et al*., 2015; Li *et al*., 2015; Lowe *et al*., 2002, 2004; Mei *et al*., 2013; Piquemal *et al*., 2005; Qu *et al*., 2012; Sampath *et al*., 2013, 2014). BLAST blastn (Camacho *et al*., 2009; task: blastn‐short, e‐value: 0.05) was used to assign positions for the forward and reverse primer sequences in the v2.1 *B. oleracea* assembly. Forward and reverse pairs not mapping on the same pseudomolecule or mapping at more than 20 positions were removed. Gene and QTL region overlap was determined using bedtools v2.27.1 intersect (Quinlan and Hall, 2010). Markers linked with *FOC1* were collected from (Lv *et al*., 2014). All marker sequences are available in Table S3. Waterfall plots were drawn using Variant Effect Predictor v88.13 (McLaren *et al*., 2016), GenVisR v1.11.3 (Skidmore *et al*., 2016), vcftools v0.1.15 (Danecek *et al*., 2011) and R 3.4.4 (R Core Team, 2016).

Alternative transcripts of Bo7g104800/*FOC1* were produced by subsetting VCF files using bcftools v1.7 view (Li, 2011), creating alternative references using GATK 3.8‐1‐0 FastaAlternateReferenceMaker (McKenna *et al*., 2010), by predicting the amino acid sequences using genometools v1.5.9 (Gremme *et al*., 2013), and then manually removing sequence after the premature stop codon. The domains of the alternative transcripts were predicted using Interproscan v5.29‐68 (Jones *et al*., 2014) using Pfam 31.0 and COILS.

## Conflict of interest

The authors have no conflicts of interest to declare.

## Supporting information


**Figure S1** Multiple sequence alignment (MUSCLE) showing all seven RLKs contained in the largest RGA candidate cluster 0.
**Figure S2** Expected (Ev_perm_) and observed (Ev_obs_) overlaps between RLK genes and PAV genes showing that the observed overlap is smaller than expected.
**Figure S3** Local *Z*‐score plot for NBS genes associated with PA based on random shuffling of positions.
**Figure S4** Expected (Ev_perm_) and observed (Ev_obs_) average distance between PAV genes and TEs.
**Figure S5** Z‐score of the association in the mean distance between PAV genes and TE genes after randomly shuffling gene positions.
**Figure S6** High impact, moderate impact and low impact SNPs per base pair compared with RGA class and presence/absence status.

**Table S1** List of *R*‐genes and their classes.
**Table S2** *P*‐values and *Z*‐scores as reported by regioneR between R‐gene candidates, PAV genes and TEs.
**Table S3** Markers linked with QTLs in the literature, their forward and reverse sequence and citation, and their position in the *B. oleracea* v2.1 TO1000DH assembly (Parkin *et al*., 2014).

## Data Availability

All data used in this study were previously published in (Golicz *et al*., 2016) and are available at http://brassicagenome.net/databases.php. Code produced for this study is available at http://github.com/appliedbioinformatics/B_oleracea_R_genes_supplementary
