# Supplementary material for: Variation in abundance of predicted resistance genes in the Brassica oleracea pangenome
Source: Plant Biotechnol J. 2018 May 31;17(4):789–800. doi: 10.1111/pbi.13015 (PMC6419861; doi:10.1111/pbi.13015)
Supplement: Supplementary file 1 — Figure S1 Multiple sequence alignment (MUSCLE) showing all seven RLKs contained in the largest RGA candidate cluster 0. Figure S2 Expected (Evperm) and observed (Evobs) overlaps between RLK genes and PAV genes showing that the observed overlap is smaller than expected. Figure S3 Local Z‐score plot for NBS genes associated with PA based on random shuffling of positions. Figure S4 Expected (Evperm) and observed (Evobs) average distance between PAV genes and TEs. Figure S5 Z‐score of the association in the mean distance between PAV genes and TE genes after randomly shuffling gene positions. Figure S6 High impact, moderate impact and low impact SNPs per base pair compared with RGA class and presence/absence status. [file PBI-17-789-s001.docx]

# Supplementary Figures

#
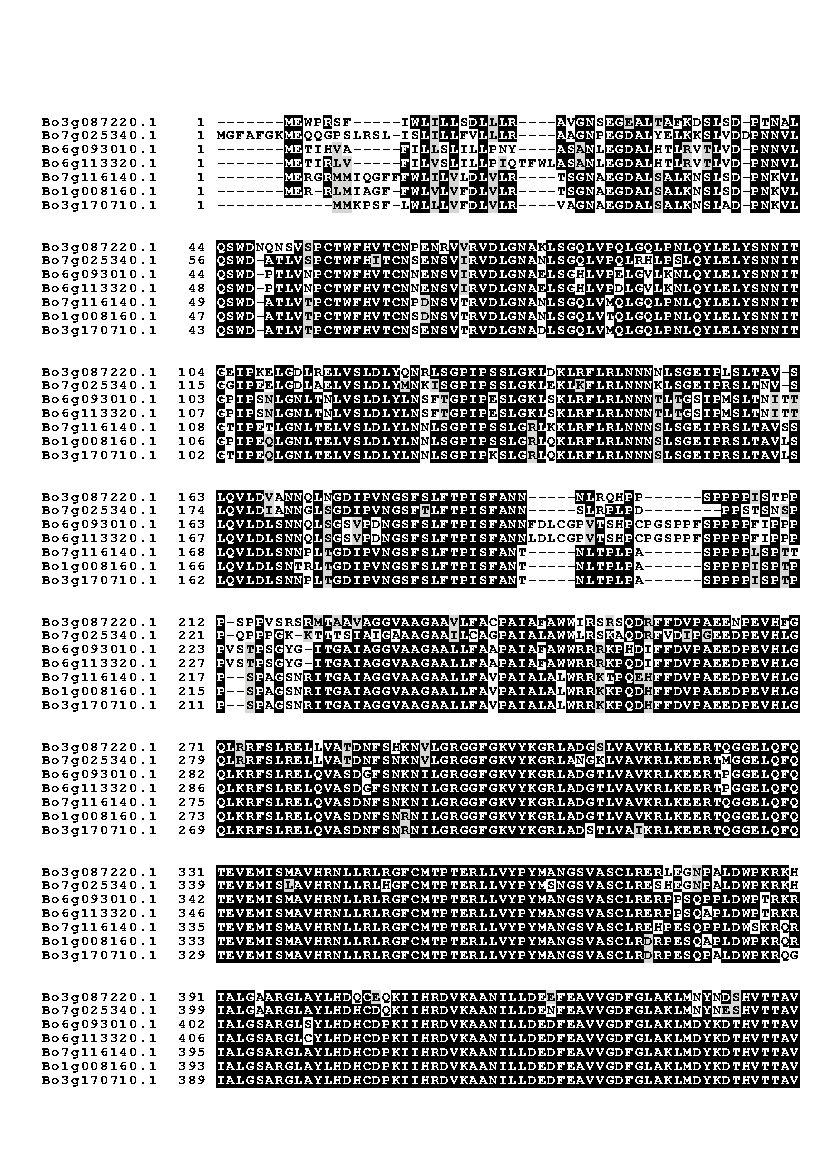


Supplementary Figure 1: Multiple sequence alignment (MUSCLE) showing all 7 RLKs contained in the largest RGA candidate cluster 0


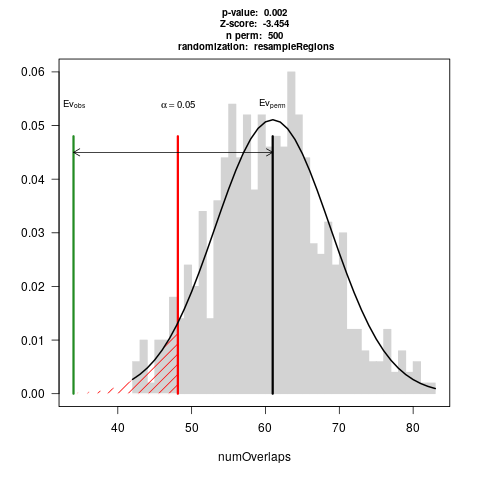


Supplementary Figure 2: Expected (Ev_perm_) and observed (Ev_obs_) overlaps between RLK genes and PAV genes showing that the observed overlap is smaller than expected


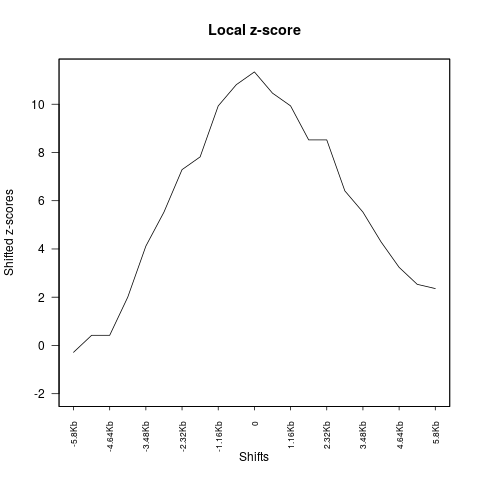


Supplementary Figure 3: Local z-score plot for NBS genes associated with PA based on random shuffling of positions


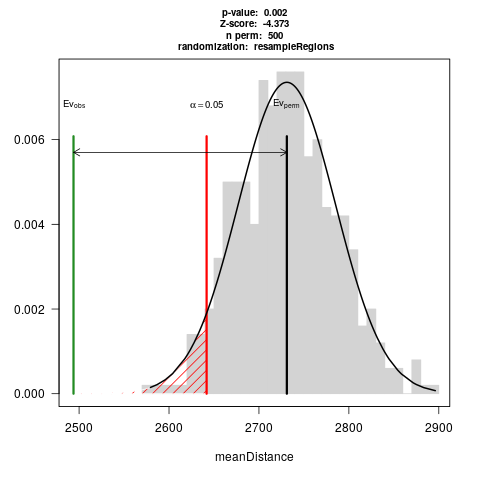


Supplementary Figure 4: Expected (Ev_perm_) and observed (Ev_obs_) average distance between PAV genes and TEs


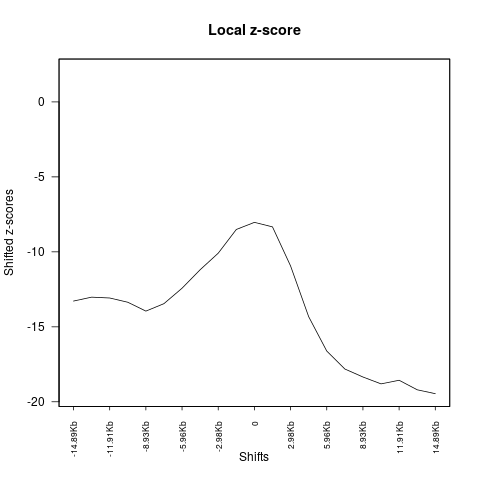


Supplementary Figure 5: Z-score of the association in the mean distance between PAV genes and TE genes after randomly shuffling gene positions


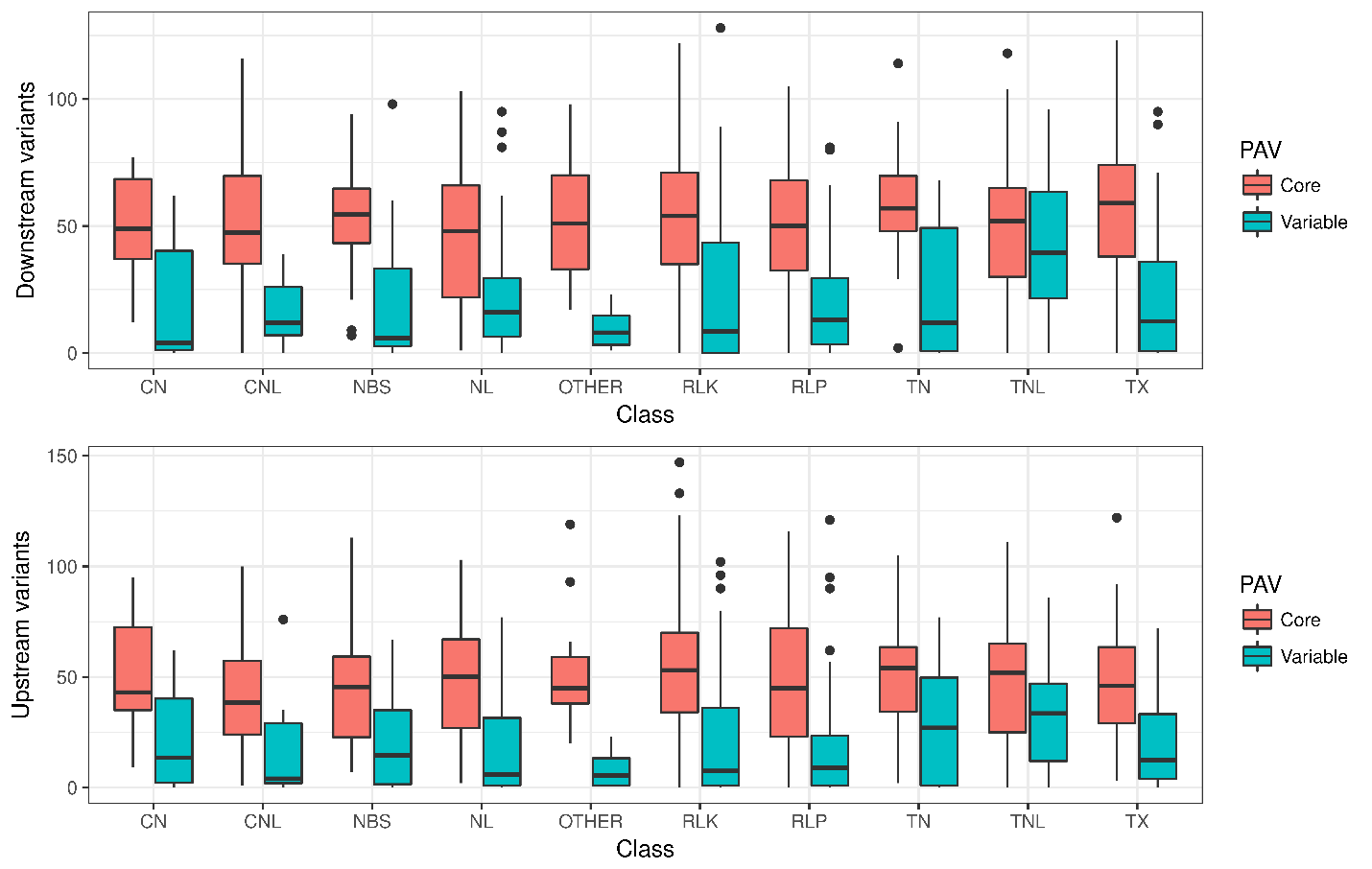


Supplementary Figure 6: High impact, moderate impact, and low impact SNPs per base pair compared with RGA class and presence/absence status
